# Rural Adolescents and Anemia: Exploring the Role of Deworming

**DOI:** 10.7759/cureus.75109

**Published:** 2024-12-04

**Authors:** Rakshit Chaudhary, Sanjeev Kumar, Seema Jain, Ganesh Singh, Neelam S Gautam, Deepika Singh

**Affiliations:** 1 Department of Community Medicine, LLRM Medical College, Meerut, IND

**Keywords:** adolescent nutrition, anemia mukt bharat, deworming, rural india, severe anemia

## Abstract

Background and objective

Anemia is one of the most common nutritional deficiency disorders worldwide. In the developing world, such as a low-to-middle-income country like India, anemia is a major public health concern. India is home to 253 million adolescents, out of which 72 million were anemic in 2018. Uttar Pradesh has the largest adolescent population in India and has twice the number of anemia cases compared to other Indian states. This study aimed to assess the prevalence of anemia in the adolescent age group, its relation to gender, and the role of deworming tablets in the prevention of anemia.

Methods

This study was conducted in a rural area of Meerut district, Uttar Pradesh. Adolescent beneficiaries of the Anemia Mukt Bharat scheme were eligible to be included. The sample size was calculated to be 170. Hemoglobin evaluation was performed using a TrueHb hemoglobinometer.

Results

The overall prevalence of anemia was 102 (60.0%). There was a higher prevalence among females (n=75, 68.2%), compared to males (n=27, 45.0%). Moderate anemia was the most common entity, affecting 69 (67.6%) participants. Among individuals who had consumed deworming tablets in the last six months, 65 (59.1%) had anemia.

Conclusions

Anemia was significantly higher among females. Three-fourths of the anemic females and half of the anemic males had moderate anemia. Deworming alone is not effective in the prevention of anemia.

## Introduction

Anemia is one of the most commonly prevalent nutritional deficiency disorders globally. It is characterized by decreased hemoglobin (Hb) concentration in relation to age, sex, altitude, and smoking status. Anemia can result from physiological factors such as pregnancy or pathological factors like increased loss or decreased production of blood or its components [1]. Globally, about half a billion women are anemic. In the developing world, such as in a low to middle-income country like India, anemia is a major public health issue [2]. Alarmingly, the prevalence of anemia in children, adolescent girls, and pregnant women is in excess of 50.0%, according to the National Family Health Survey-5 (NFHS-5) (2019-21). The prevalence is higher in rural areas compared to urban areas [3,4].

Adolescence is a period of rapid growth and development characterized by major physiological and biochemical changes in the body. In terms of nutrient requirement, it is a phase of increased demand. In both adolescent girls and boys, increased iron uptake is required to meet a rapid growth spurt; also, in girls, further supply is required to compensate for menstrual blood loss. Worldwide, one in four individuals aged 10-24 years suffers from anemia, which amounts to 430 million individuals. India is considered a nation of youth, and it is home to 253 million adolescents, out of which 72 million were anemic in 2018. Uttar Pradesh has the largest adolescent population in India and has twice the number of anemia cases compared to other Indian states [5].

According to WHO, anemia is defined as a Hb concentration of less than 13 g/dL in males (15 years and above) and less than 12 g/dL in non-pregnant females (15 years and above) [6]. Anemia in adolescents may lead to decreased growth and productivity and low scholastic performance, and, in the long term, can cause cardiomegaly and left ventricular failure. Additionally, anemia also causes complications related to pregnancy and childbirth in young females. This study was planned to assess the prevalence of anemia in the adolescent age group, as well as to examine the association between the prevalence of anemia and the sex of the participants, and the role of deworming tablets in preventing anemia.

## Materials and methods

A community-based cross-sectional study was conducted in a rural area of Meerut district from September to December 2023. The study population involved adolescent beneficiaries of the Anemia Mukt Bharat program in the Behsuma area. The inclusion criteria were as follows: all adolescent health beneficiaries of the Anemia Mukt Bharat program. Those not willing to participate or those suffering from any chronic debilitating illness were excluded from the study The sample size was calculated based on the following formula: N = $\frac{Z^2_{\alpha/2} \times p \times q}{d^2}$; based on the NFHS-5 data for the prevalence of anemia in Meerut district (45.1%), and at 7.5% absolute precision, the required sample size came out to be 170.

A simple random sampling method was employed for data collection. Out of 31 rural primary health centers (PHC) in Meerut district, one PHC was randomly selected using the lottery method, which turned out to be PHC Behsuma. The first household adjacent to the PHC was selected using the pencil tip method, and then subsequent households towards the left of the first house were considered until the desired sample size was reached.

Data were collected using a pre-designed and pre-tested questionnaire (see Appendices). Consent and assent were obtained from the guardians and beneficiaries, respectively, after explaining the nature of the study. A capillary blood sample was taken after cleaning and drying the index or ring finger of the non-dominant hand. Hb estimation was performed using a True-Hb hemoglobinometer (Wrig Nanosystems, New Delhi, India). WHO classification of anemia was used to classify beneficiaries. Data were tabulated and analyzed using Microsoft Excel. Chi-square was used as the test of statistical significance, and a p-value less than 0.05 was considered significant. Ethical approval was obtained from the institutional ethical committee (approval no: No./SC-1/2024/5716).

## Results

Out of the 170 respondents, 60 (35.3%) were males, while 110 (64.7%) were females. Of note, 102 (60.0%) of the total respondents were found to be anemic, while 68 (40.0%) were non-anemic. Among males, 27 (45.0%) were anemic, whereas among females, 75 (68.2%) had anemia, as shown in Table 1. There was a significant association between the sex of the participant and the prevalence of anemia, as females were more anemic compared to males (p=0.0032).

**Table 1:** Association between anemia incidence and sex of the participant (N=170) *P-value less than 0.05 suggests statistical significance

Sex of participant	Anemia			P-value
	Present	Absent	Total	
	N (%)	N (%)	N (%)	0.0032*
Male	27 (45.0)	33 (55.0)	60 (35.3)	
Female	75 (68.2)	35 (31.8)	110 (64.7)	
Total	102 (60.0)	68 (40.0)	170 (100)	

Among anemic individuals, moderate anemia was the most common entity, affecting 69 (67.6%) participants, followed by mild anemia in 20 (19.6%) and severe anemia in 13 (12.8%) individuals. Amongst anemic males and females, moderate anemia was present in 14 (51.9%) and 55 (73.3%) participants, respectively. Mild and severe anemia was present in eight (29.6%) and five (18.5%) anemic males, and 12 (16.0%) and eight (10.7%) anemic females respectively. The association between the sex of the participant and the severity of anemia was not significant (p=0.123), as indicated in Table 2.

**Table 2:** Association between sex of the individual and severity of anemia (n=102)

Sex	Severity of anemia			Total	P-value
	Mild	Moderate	Severe		0.123
	N (%)	N (%)	N (%)	N (%)	
Male	08 (29.6)	14 (51.9)	05 (18.5)	27 (26.5)	
Female	12 (16.0)	55 (73.3)	08 (10.7)	75 (73.5)	
Total	20 (19.6)	69 (67.6)	13 (12.8)	102 (100)	

Among the 170 participants, 110 (64.7%) had consumed deworming tablets in the last six months, while 60 (35.3%) had not. As shown in Table 3, we observed a slightly lower prevalence of anemia (n=65, 59.1%) in participants who had consumed deworming tablets as compared to those who had not consumed deworming tablets (n=37, 61.6%). The findings were not significant (p=0.74). Among 102 anemic participants, 65 (63.7%) had consumed deworming tablets in the last six months, while 37 (36.3%) had not. Among anemic individuals who had consumed deworming tablets, mild, moderate, and severe anemia was present in 13 (20.0%), 45 (69.2%), and seven (10.8%), respectively; in anemic individuals who had not taken deworming tablets, seven (18.9%), 24 (64.9%) and six (16.2%) had mild, moderate, and severe anemia respectively. Although the findings were not significant (p>0.05), severe anemia was more common in those who had not consumed deworming tablets in the last six months, as demonstrated in Table 4.

**Table 3:** Association between deworming and anemia (N=170)

Consumption of deworming tablet	Anemia			P-value
	Present	Absent	Total	
	N (%)	N (%)	N (%)	0.74
Yes	65 (59.1)	45 (40.9)	110 (64.7)	
No	37 (61.6)	23 (38.4)	60 (35.3)	
Total	102 (60.0)	68 (40.0)	170 (100)	

**Table 4:** Association between deworming and severity of anemia (n=102)

Consumption of deworming tablet	Severity of anemia			Total	P-value
	Mild	Moderate	Severe		
	N (%)	N (%)	N (%)	N (%)	0.73
Yes	13 (20.0)	45 (69.2)	07 (10.8)	65 (63.7)	
No	07 (18.9)	24 (64.9)	06 (16.2)	37 (36.3)	
Total	20 (19.6)	69 (67.6)	13 (12.8)	102 (100)	

## Discussion

The present study revealed the overall prevalence of anemia in the adolescent age group to be 102 (60.0%), which is slightly higher compared to NFHS-5 data for rural areas of Uttar Pradesh. This study has shown a higher prevalence of anemia among females (n=75, 68.2%) compared to males (n=27, 45.0%). There was a significant association between the sex of the participant and the prevalence of anemia (p<0.05). Similarly, NFHS-5 [4] data for rural areas of Uttar Pradesh revealed a higher prevalence of anemia in non-pregnant females (15-49 years), i.e., 50.7% as compared to males (15-19 years), in whom the prevalence of anemia was 29.9%. Kishore et al.'s [7] study conducted in Rishikesh (Uttarakhand) showed that the overall prevalence of anemia was 53.2%, out of which 45.1% were males and 54.6% were females. The increased prevalence of anemia in females can be due to increased physiological demand, menstrual blood loss, poor eating habits in females, as well as social neglect.

In the present study, moderate anemia was the most common type, affecting 69 (67.6%) of the anemic individuals, whereas mild and severe anemia was present in 20 (19.6%) and 13 (12.8%), respectively. Similar findings were reported by Bajaj et al. [8] (2022) from Pune, Maharashtra, where moderate anemia was the most prevalent form, affecting 55.0% of the study participants. This was followed by mild anemia at 40.7% and severe anemia at 4.3%. Among anemic males, mild, moderate, and severe anemia was present in eight (29.6%), 14 (51.9%), and five (18.5%), respectively; among anemic females, the prevalence was 12 (16.1%), 55 (73.3%), and eight (10.7%) respectively. Moderate anemia was more common in females, while mild and severe anemia was common in males. Kishore et al. [7] (2020) reported that moderate and severe anemia were more common in females than males, whereas mild anemia was common in males. The reason for this prevalence of moderate and mild anemia could be that moderate anemia can go unnoticed for long periods with nonspecific symptoms like weakness and fatigue while severe anemia causes more pronounced symptoms like dyspnea on slight exertion or even at rest and hence is treated promptly in clinical settings. 

In this study, 110 (64.7%) children had consumed deworming tablets in the last six months and they exhibited a slightly lower prevalence of anemia: (n=65, 59.1%) as compared to those who had not consumed (n=37, 61.6%). However, the difference was not statistically significant (p>0.05). This shows that deworming alone is not effective in anemia prevention. The most probable reasons for ineffective deworming could be low nutritional intake, increased iron demand in the adolescent age group, recurrent worm infestations in rural settings, and poor service utilization. Scott et al. [5] reported that 25.8% of children in their study had consumed deworming tablets in the last six months and that deworming tablets are an effective intervention in anemia prevention in southern India (OR: 1.77).

Kamble et al. [9] reported that anemia prevalence among adolescent girls who received albendazole tablets for deworming on National Deworming Day was significantly lower as compared to those who did not undergo deworming (p<0.001). Alamneh et al.'s [10] study from Ethiopia among different age groups (children aged 6-59 months) reported that deworming was effective in reducing moderate anemia by 21.0%, but its effect on mild and severe anemia was not significant. Severe anemia was reported in a lower proportion of participants (n=7, 10.8%) who had a history of deworming in the last six months compared to those who had not undergone deworming (n=6, 16.2%), although the difference was not significant (p>0.05). There is a paucity of research on the effects of deworming in reducing the severity of anemia.

Limitations of the study

This study has a few limitations. Primarily, it had a relatively smaller sample size, which may make generalizing the results to the broader population challenging. Moreover, calibrating and recalibrating the instruments may have affected the results - a lower proportion of the male population in the study.

## Conclusions

The present study included adolescent beneficiaries (aged 10-19 years) of the Anemia Mukt Bharat program in a rural area of Meerut, Uttar Pradesh. About two-thirds of the study population were females. More than half of the study participants were anemic, and anemia was significantly higher in females compared to males. This can be attributed to increased menstrual blood loss, selective food habits among females, and, perhaps, ignorance due to gender discrimination. Among anemic participants, more than two-thirds were moderately anemic. The association between deworming and the prevalence of anemia was not significant. Among the anemic population, severe anemia was more common in those who had not consumed deworming tablets, but the results were not significant. Deworming alone can neither effectively prevent anemia nor decrease its severity significantly. Dietary patterns, nutritional deficiencies, vitamin C supplementation, and other non-nutritional causes of anemia need to be addressed promptly and extensively.

## Appendices

**Table 5:** Questionnaire employed in the study

S. no.	Rural Adolescents and Anemia: Exploring the Relationship with Deworming	Response
1	Name:	
2	Age:	
3	Gender: male/female	
4	Religion: Hindu/Muslim/Sikh/Christian/others	
5	Total monthly family income:	
6	Number of family members:	
7	Birth order of participant:	
8	Soci-economic status:(Modified BG Prasad Classification April 2023): Class: I/II/III/IV/V	
9	Type of house: Kucha/Pucca/semi-Pucca	
10	Signs/symptoms of anemia: generalized weakness/fatigue/pallor/koilonychia	
11	Hemoglobin percentage: ______________ gram/deciliter (using True-Hb hemoglobinometer)	
12	Type of anemia: mild/moderate/severe (as per WHO criteria)	
13	Deworming tablet taken in last six months: yes/no	
14	If yes (from where): public health facility/school health authorities/private practitioners	
